# Supplementary material for: In Vitro Functional Validation of an Anti-FREM2 Nanobody for Glioblastoma Cell Targeting
Source: Antibodies (Basel). 2025 Jan 24;14(1):8. doi: 10.3390/antib14010008 (PMC11843905; doi:10.3390/antib14010008)
Supplement: Supplementary file 1 [file antibodies-14-00008-s001.zip › Supplementary file 2 copy.pdf]

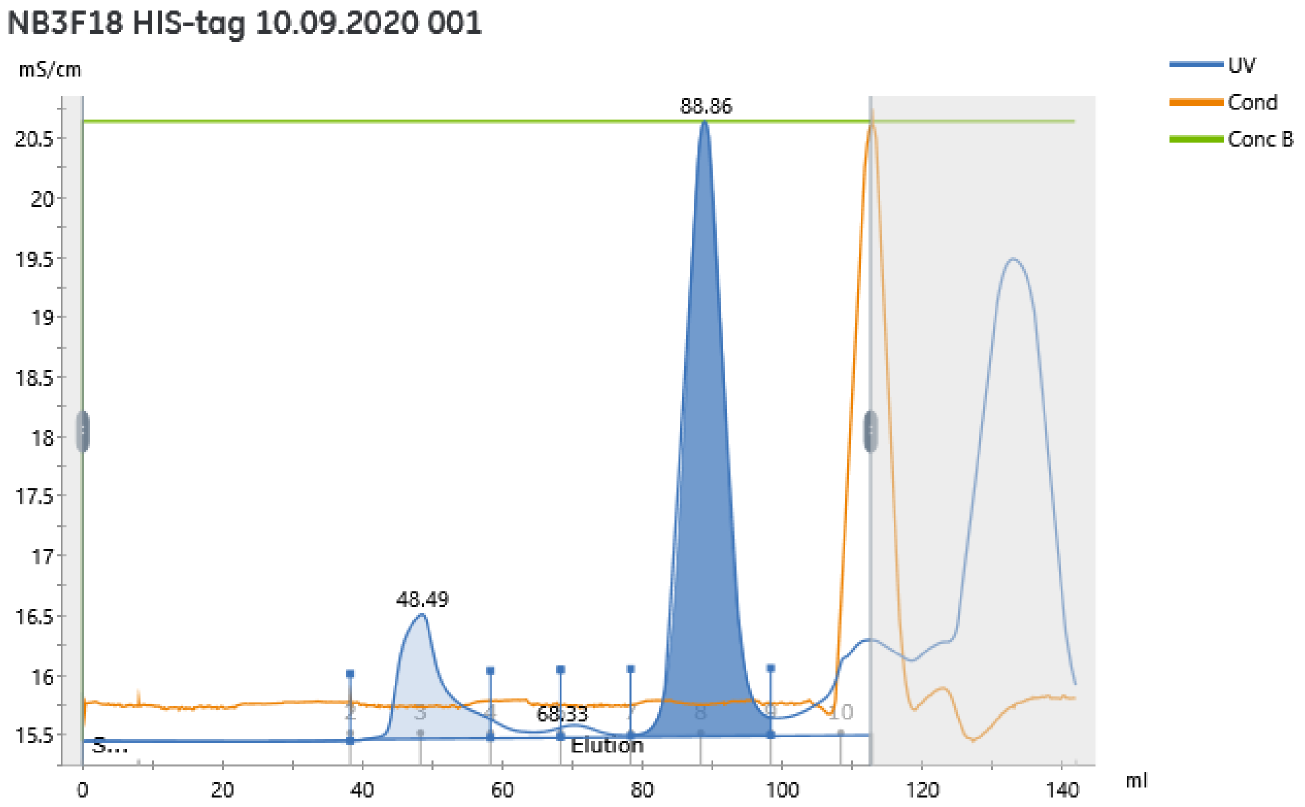

Peak Table

Peak	Retention (ml)	Area (ml*mAU)	Area %	Ext coeff. (mg ml $^{-1}$ cm $^{-1}$)	Fraction(s)	Volume (ml)
Peak A	48.494	221.0	18.63		2 - 3	20.009
Peak B	68.330	0.1083	0.01		5	0.049
Peak C	88.861	965.4	81.36	1.476	7 - 8	20.002

Amount (mg)	Concentration (mg/ml)	Conductivity (mS/cm)
		15.75
		15.75
3.270	0.163	15.77

Created 9/10/2020 9:39:11 AM +02:00 by Default on AKTApure
Printed 9/10/2020 12:05:13 PM +02:00 by Default
\DefaultHome\Results\
