# Supplementary figures and images for: In Vitro Functional Validation of an Anti-FREM2 Nanobody for Glioblastoma Cell Targeting
Source: Antibodies (Basel). 2025 Jan 24;14(1):8. doi: 10.3390/antib14010008 (PMC11843905; doi:10.3390/antib14010008)

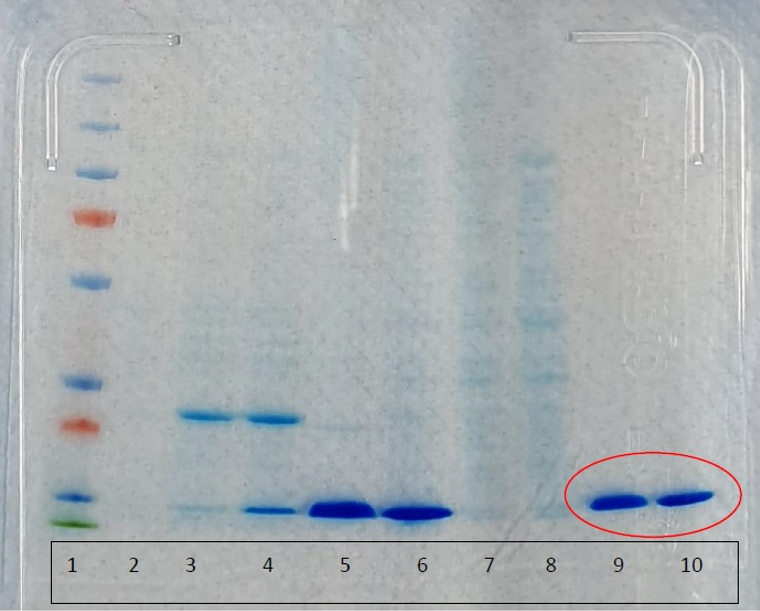

Supplement: Supplementary file 1 [file antibodies-14-00008-s001.zip › Supplementary file 3 copy.png]

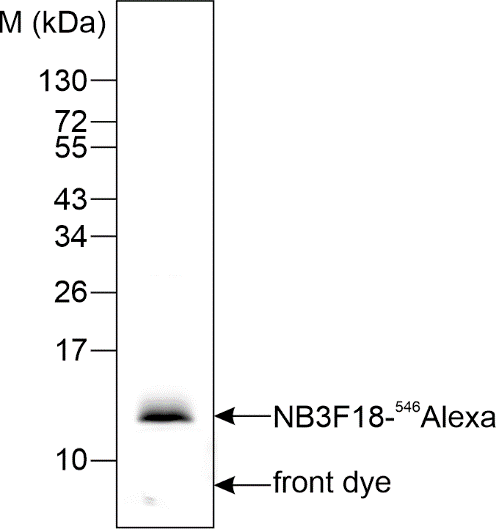

Supplement: Supplementary file 1 [file antibodies-14-00008-s001.zip › Supplementary file 4 copy.png]

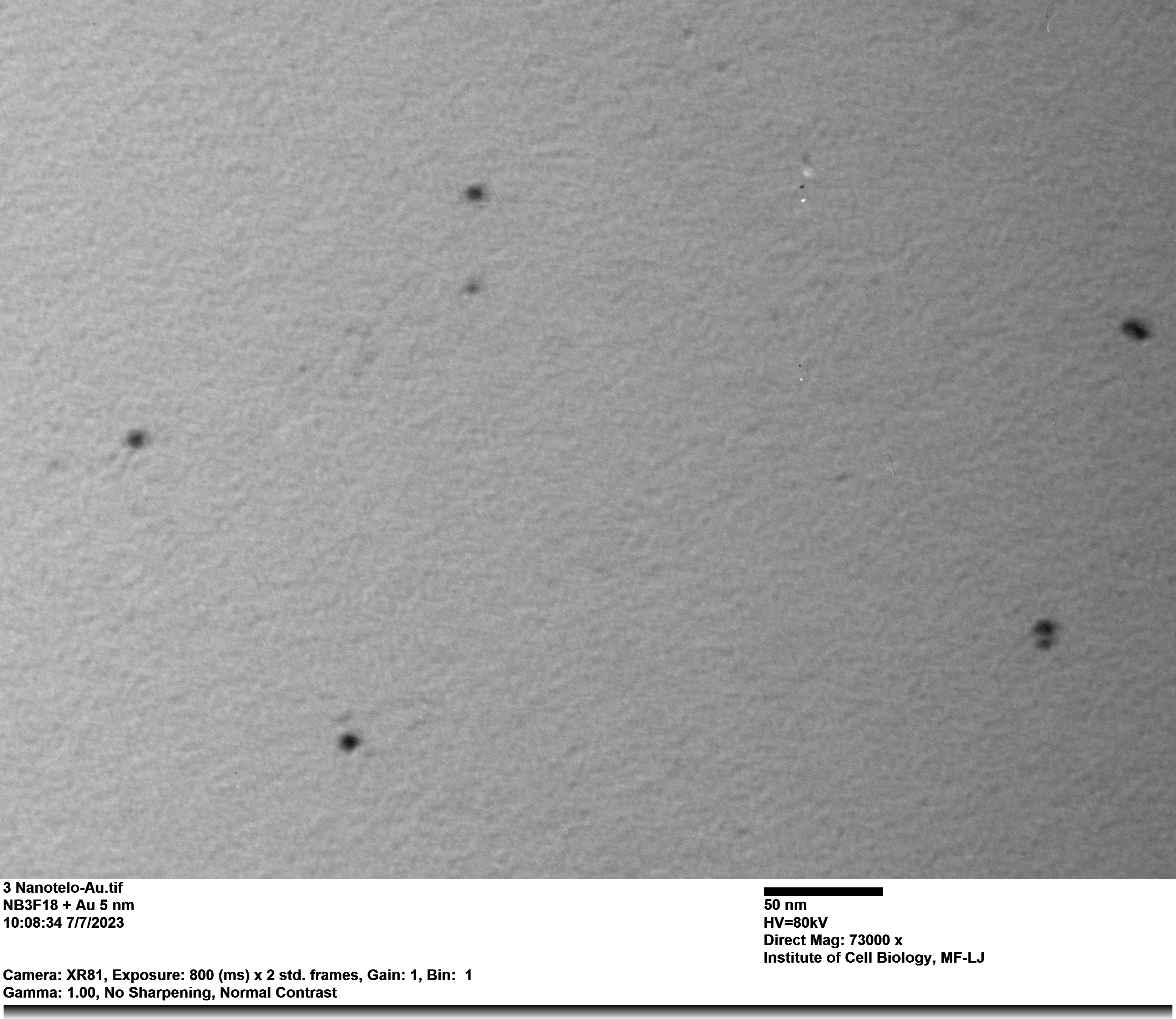

Supplement: Supplementary file 1 [file antibodies-14-00008-s001.zip › Supplementary file 5 copy.TIF]
